# *A posteriori* determination of the useful data range for small-angle scattering experiments on dilute monodisperse systems

**DOI:** 10.1107/S2052252515005163

**Published:** 2015-04-21

**Authors:** Petr V. Konarev, Dmitri I. Svergun

**Affiliations:** a Hamburg Outstation, European Molecular Biology Laboratory, Notkestrasse 85, Hamburg 22607, Germany; b Laboratory of Reflectometry and Small-angle Scattering, Institute of Crystallography of the Russian Academy of Sciences, Leninsky prospekt 59, Moscow 119333, Russian Federation

**Keywords:** small-angle scattering, WAXS, SAXS, solution scattering, protein structure, *Shanum*

## Abstract

Small-angle X-ray and neutron scattering (SAXS and SANS) experiments on solutions provide rapidly decaying scattering curves, often with a poor signal-to-noise ratio, especially at higher angles. On modern instruments, the noise is partially compensated for by oversampling, thanks to the fact that the angular increment in the data is small compared with that needed to describe adequately the local behaviour and features of the scattering curve. Given a (noisy) experimental data set, an important question arises as to which part of the data still contains useful information and should be taken into account for the interpretation and model building. Here, it is demonstrated that, for monodisperse systems, the useful experimental data range is defined by the number of meaningful Shannon channels that can be determined from the data set. An algorithm to determine this number and thus the data range is developed, and it is tested on a number of simulated data sets with various noise levels and with different degrees of oversampling, corresponding to typical SAXS/SANS experiments. The method is implemented in a computer program and examples of its application to analyse the experimental data recorded under various conditions are presented. The program can be employed to discard experimental data containing no useful information in automated pipelines, in modelling procedures, and for data deposition or publication. The software is freely accessible to academic users.

## Abstract 2

The useful experimental data range for solution small-angle scattering is assessed through the number of meaningful Shannon channels that can be determined from a given data set, and a robust algorithm is provided to determine this range automatically.

## 1. Introduction   

Small-angle scattering (SAS) of X-rays (SAXS) and neutrons (SANS) is a powerful method for the analysis of biological macromolecules in solution (Svergun *et al.*, 2013 ▶). Over the last decade, major advances in instrumentation and computational methods have led to new and exciting applications of SAXS to structural biology (Graewert & Svergun, 2013 ▶). However, for biological systems the contrast of the particles in aqueous solution is rather small and the useful signal may be weak compared with the background (Jacques *et al.*, 2012 ▶). This leads to a low signal-to-noise ratio for the data, especially at higher scattering angles. A question arises as to how to determine the useful angular data range of the experimental scattering pattern that can be taken for subsequent interpretation and model building. A common practice is to use only that portion of the scattering curve where the signal-to-noise ratio exceeds a certain threshold (Skou *et al.*, 2014 ▶), but the choice of the threshold remains a rather subjective procedure. Also, relying only on the signal-to-noise ratio does not take into account the degree of oversampling of the data.

The problem of assessing the useful data range is also pertinent for other diffraction techniques, *e.g.* X-ray crystallography. Accepted criteria for data quality and accuracy include the signal-to-noise ratio of the intensities in the highest resolution shell [〈*I*/σ(*I*)〉] and the spread function of the equivalent reflections (*R*_merge_) (Wlodawer *et al.*, 2008 ▶). In SAS data analysis, no agreed criteria exist and, in view of the recent standardization developments of SAS publications (Jacques *et al.*, 2012 ▶; Trewhella *et al.*, 2013 ▶) and efforts towards making experimental data and models publicly available (Valentini *et al.*, 2015 ▶), the absence of an objective method to assess the useful range of a data set is a serious drawback.

Here, we present an approach using Shannon sampling (Shannon & Weaver, 1949 ▶) to determine the useful range in a given experimental scattering data set from a dilute monodisperse system *via* the number of Shannon channels that can be determined from this data set. To establish a robust algorithm for the determination of this number, simulated data sets with different signal-to-noise ratios and different oversampling corresponding to typical X-ray and neutron scattering experiments are generated and analysed. The algorithm is implemented in a computer program and applied to experimental SAXS and SANS data sets recorded under various conditions and on various instruments. The proposed method is easy to incorporate into automated analysis pipelines, and it can also be employed to select a fitting range in modelling procedures, especially those relying on higher resolution data, and during data deposition or publication to discard the portions of the (higher-angle) SAS data containing no useful information.

## 2. Truncated Shannon approximation   

The scattering intensity *I*(*s*) from a dilute solution of identical particles (*e.g.* a monodisperse solution of macromolecules) is related to the distance distribution function *p*(*r*) in real space as
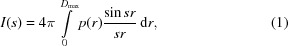
where *s* = 4πsin(θ)/λ, 2θ is the scattering angle and λ is the radiation wavelength. Equation (1) takes into account the fact that the *p*(*r*) function has finite support and it is equal to zero for all *r* > *D*_max_ (where *D*_max_ is the maximum size of the particle). If *I*(*s*) is known, *p*(*r*) can be calculated by the inverse transformation
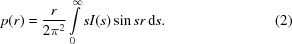
From equations (1) and (2), one can easily see that the functions *sI*(*s*) and *p*(*r*)/*r* are Fourier mates related by a sine transformation, and that *p*(*r*) is conveniently represented as a Fourier sine series
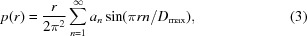
where *n* is an integer. Substituting equation (3) into equation (1) gives the Shannon interpolation formula (Shannon & Weaver, 1949 ▶) 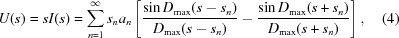
where *s*_*n*_ = *n*π/*D*_max_ are the positions of the Shannon channels.

Equation (4) contains, generally speaking, an infinite number of Shannon channels. However, for experimental data measured over a limited range of scattering vectors (*s* < *s*_max_), the contribution of the channels beyond this range (*i.e.* with indices *n* > *s*_max_ *D*_max_/π) to the fit in this range is relatively small. The number of Shannon channels in the measured range, *N*_S_ = *s*_max_ *D*_max_/π, was therefore suggested (Damaschun *et al.*, 1968 ▶; Taupin & Luzzati, 1982 ▶) as an estimate of the information content of the scattering data. Methods have been proposed to calculate the *p*(*r*) function (Moore, 1980 ▶) and to assess fits to experimental data (Rambo & Tainer, 2013 ▶) based on the Shannon representation.

Although larger values of *N*_S_ do generally indicate a greater information content, it is clear that this value alone cannot provide an ultimate estimate, due to the fact that the signal-to-noise ratio is not taken into account. Furthermore, SAS data are usually oversampled, *i.e.* measured with an angular increment Δ*s* much smaller than the distance between the Shannon channels π/*D*_max_. The amount of information in the data must be related to both the level of experimental error and the degree of oversampling.

When the summation index in equations (3) and (4) is limited by an integer number *M*, the corresponding truncated expressions are denoted *p*_*M*_(*r*) and *U*_*M*_(*s*), respectively. Given an experimental data set, one can construct its truncated approximation *U*_*M*_(*s*) using *M* Shannon channels by minimizing the discrepancy
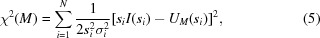
where the summation index *i* runs over *N* experimental points and 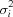 is the standard deviation for the measured intensity at *s*_*i*_. The best least-squares solution should meet the condition δχ^2^/δ*a*_*m*_ = 0, leading to the system of normal equations
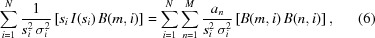
where 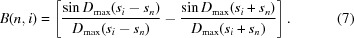



For solution scattering experiments, the experimental data *I*(*s*_*i*_) represent the difference between the scattering from the solute and the pure solvent, and may show negative values due to experimental errors. These negative values should enter equations (5) and (6). However, the computed SAS intensity *U*_*M*_(*s*_*i*_) must always be non-negative, and equation (6) can be solved using standard methods under the constraint of non-negativity of *a*_*n*_ (Lawson & Hanson, 1974 ▶).

The truncated Shannon approximation provides a way of assessing the information content and useful range of an experimental data set. Indeed, if *M* is too small, this approximation will not have a sufficient number of terms to fit the experimental data. With increasing *M* one will improve the fit, but at some stage an overfitting would be observed where the determined *a*_*n*_ values will not significantly improve the discrepancy, being poorly defined by the experimental data. There should therefore be an optimum (effective) value of the channels *M*_S_ reflecting the information content of the data, and the useful range of the given experimental data set will be defined as π*M*_S_/*D*_max_. Note that *M*_S_ does not necessarily coincide with *N*_S_, and the following sections will present a procedure for a reliable automated determination of the effective number of Shannon channels.

## 3. Noise level and oversampling   

In order to test how the truncated Shannon approximation is influenced by noise and oversampling, we have simulated a number of scattering patterns from various geometric bodies (see Table 1 ▶). The data were generated with a fixed momentum transfer value up to *s*_max_ = 4 nm^−1^ and containing varying numbers of Shannon channels for different bodies due to their different size. A dense grid with an angular step Δ*s* = 0.0025 nm^−1^ was used to simulate typical synchrotron X-ray data collection, and a sparse grid with Δ*s* = 0.042 nm^−1^ (*i.e.* having about 17 times fewer points in the same angular range) emulated SANS data. For each intensity point, random Gaussian noise was added, with the relative error of the simulated noise varying from 1 to 400% for the different data sets.

**Figure 1:**
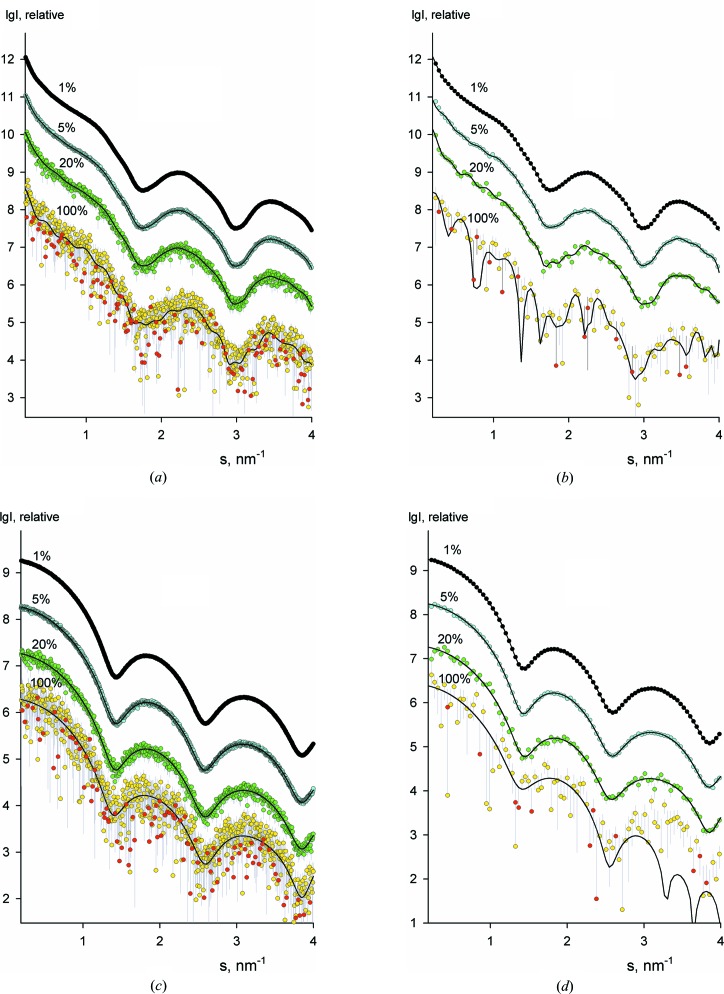
Simulated scattering curves from an oblate ellipsoid [half-axes 1, 15 and 15 nm, parts (*a*) and (*b*)] and for a cube with an edge of 5 nm [parts (*c*) and (*d*)]. From top to bottom, the curves correspond to added Gaussian noise of 1, 5, 20 and 100% (dots with error bars), respectively. The best truncated Shannon approximations are displayed as solid lines. Subsequent curves are shifted by one logarithmic order for better visualization. Here and in the subsequent figures, the intensities are displayed on a logarithmic scale. For the noisy simulated and experimental data where the values may become negative because of noise, logarithms of the modulus of the intensity are displayed as red dots. Parts (*a*) and (*c*) correspond to X-ray type data, and parts (*b*) and (*d*) to neutron type data.

For each simulated data set, Shannon fits were calculated with increasing *M* according to equations (4)–(6), and the quality of the approximation was assessed by the *R* factor between the ideal theoretical curve without noise *I*_ref_(*s*) and the corresponding Shannon fit *U*_*M*_(*s*)/*s*, according to the formula
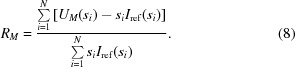
The simulated data sets and the best Shannon fits (corresponding to the minimum *R* factors) are shown in Fig. 1 ▶ and in the supporting information (Figs. S1–S4). The optimum number of Shannon channels *M_B_* providing the best agreement with the ideal curve depends on both the noise level and the angular step (see Table 1 ▶). One should also note that the quality of the fits from the truncated Shannon approximation depends on the anisometry of the object. For very anisometric particles, high noise levels (100% noise in Fig. 1 ▶ *a*; 20 and 100% noise in Fig. 1 ▶ *b*) lead to significant oscillations in the Shannon approximations. Still, all the fits in Fig. 1 ▶, even those with oscillations, provide the best agreement with the ideal curve compared with the Shannon fits with other *M*, and are therefore best fits in terms of the truncated Shannon approximation.

As is evident from Table 1 ▶(*a*), for oversampled and accurate (1–5% noise) data the best Shannon fits sometimes require more channels *M_B_* than *N*_S_, indicating that the amount of information in the data warrants extrapolation beyond the available range. This possibility reflects the well known property of oversampled measurements of analytical functions [and the scattering intensity, being a Fourier transform of a *p*(*r*) function having a finite support, is an analytical function according to the Wiener–Paley–Schwartz theorem (Schwartz, 1952 ▶)]. The effect is utilized *e.g.* for ‘super-resolution’ in optical image reconstruction (Frieden, 1971 ▶) but can clearly be observed only for very accurate data. Obviously, *M_B_* decreases with an increasing level of added noise, but interestingly and somewhat unexpectedly, for oversampled data, even at a very high (100% and above) noise level, *M_B_* may still be essentially equal to *N*_S_ (taking into account the ±1 uncertainty of determination of *M_B_*). In other words, oversampled data, even if looking very noisy (*e.g.* Fig. 1 ▶ *c*, bottom curve), still contain useful information about the ideal scattering curve over the entire measured range. In contrast, for data simulated on a sparse angular grid, *M_B_* starts to decrease at a noise level of 20–50% (Table 1 ▶ *b*), indicating an insufficient quantity of information to define *N*_S_ channels for sparse noisy data.

## 4. Determination of the effective number of Shannon channels   

In a real experiment, the ideal scattering curve and thus *M_B_* are of course not available, and *M*_S_ should be determined based on experimental data only. The extensive simulations described in the previous section allowed us to define quantitative criteria for the selection of *M*_S_. In principle, the choice could be performed by monitoring the discrepancy χ^2^ of the Shannon fit as a function of *M*, given that the poorly defined channels would not significantly improve the fit. Such a procedure is employed to determine the number of independent components in singular-value decomposition (Golub & Reinsch, 1970 ▶), although formalization of the ‘non-significant’ condition is not trivial and the results are not always accurate. Fortunately, a reliable estimate of *M*_S_ is obtained by combining reciprocal- and real-space criteria. Indeed, each Shannon approximation *U*_*M*_(*s*) expressed by a set of coefficients *a*_*n*_ corresponds to a distance distribution in real space *p*_*M*_(*r*) according to equation (3). Increasing *M* adds extra terms to *p*_*M*_(*r*), oscillating with a higher and higher frequency π*M*/*D*_max_. One would expect that the unreliably determined Shannon channels *a*_*n*_ will provide nothing but increasing oscillations in the *p*_*M*_(*r*) function, and this can be captured by a measure of the integral derivative Ω(*p*)
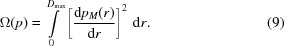
The quality of the Shannon representation can be characterized by a combined measure
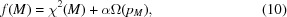
where the coefficient α ensures proper scaling of the two metrics (see below). The procedure to determine the optimum number of Shannon channels *M*_S_ is therefore formulated as follows:

(i) Given an experimental data set, estimate the maximum particle size *D*_max_ (this is done *e.g.* by the programs *AutoRG* and *AutoGnom* (Petoukhov *et al.*, 2007 ▶).

(ii) Calculate the nominal number of Shannon channels as *N*_S_ = *s*_max_ *D*_max_/π, and set up the search range. In practical applications, we use *M*_min_ = max(3, 0.2*N*_S_), *M*_max_ = 1.25*N*_S_.

(iii) For *M*_min_ < *M* < *M*_max_, calculate the coefficients of the Shannon approximation *a*_*n*_ (*n* = 1, … *M*) by solving equation (6) using a non-negative linear least-squares procedure (Lawson & Hanson, 1974 ▶).

(iv) For each Shannon fit, calculate the discrepancy χ^2^(*M*) and the integral derivative Ω(*p*_*M*_).

(v) Evaluate the scaling coefficient α as the ratio between χ^2^(*M*_max_) and Ω[*p*(*M*_min_)].

(vi) Determine the optimum value *M*_S_ corresponding to the minimum of the target function *f*(*M*) as defined in equation (10).

**Figure 2:**
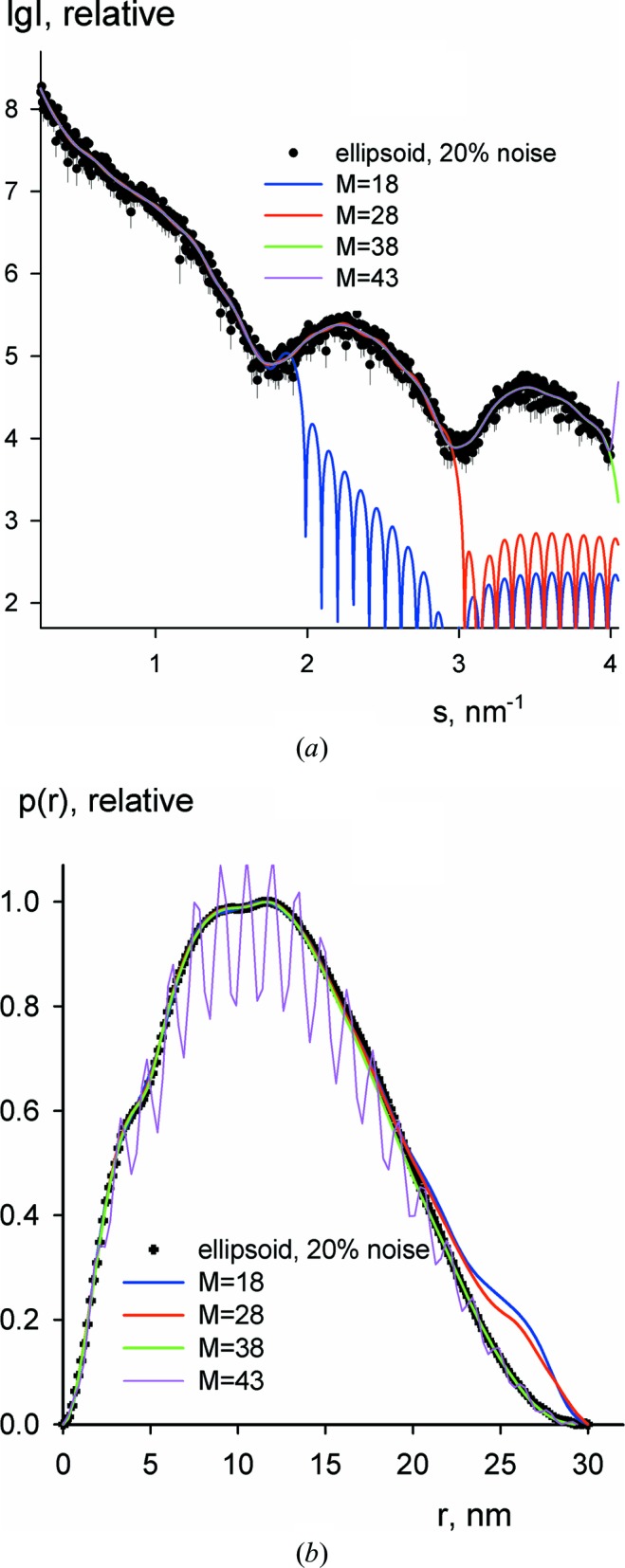
Truncated Shannon fits for simulated scattering from an oblate ellipsoid (half-axes 1, 15 and 15 nm) with added 20% noise. (*a*) The scattering pattern with noise (dots with error bars) and the Shannon approximations obtained using *M* = 18 (blue curve), 28 (red curve), 38 (green curve) and 43 (pink curve). (*b*) The distance distribution function *p*(*r*) calculated from the noise-free simulated data (dots) and *p*_*M*_(*r*) from the appropriate Shannon approximations (coloured curves). The colour scheme is the same as in part (*a*).

**Figure 3:**
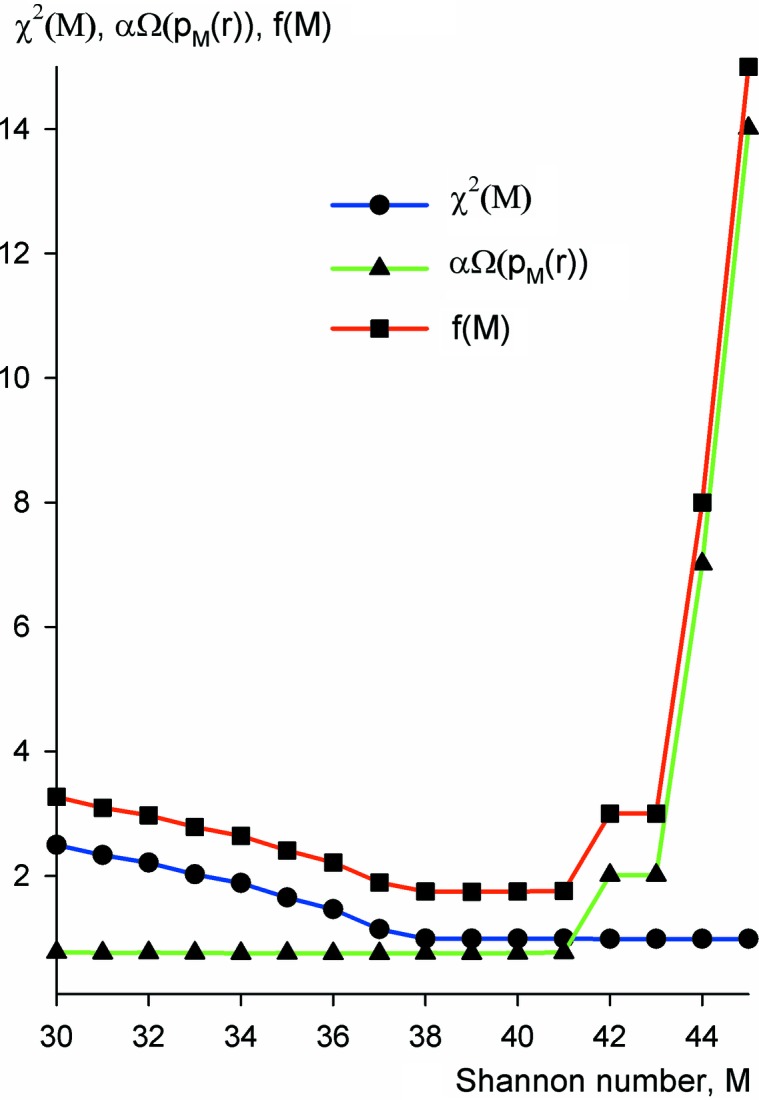
Discrepancy and smoothness as a function of the number of Shannon channels for the simulated data in Fig. 2 ▶. The blue curve depicts the discrepancy χ^2^(*M*) and the green curve the scaled integral smoothness αΩ[*p*_*M*_(*r*)]. The target function *f*(*M*) is shown as the red curve.

Typical examples of fits with different numbers of Shannon channels and the corresponding *p*(*r*) functions are shown in Figs. 2 ▶(*a*) and 2 ▶(*b*) for the case of an oblate ellipsoid. As expected, the χ^2^ values decrease with increasing Shannon channel number (Fig. 3 ▶, blue curve), reaching a plateau when approaching *M_B_* (which, for this example, coincides with *N*_S_). The integral derivative Ω(*p*_*M*_) increases slightly with increasing *M* and displays a sharp upturn when *M* exceeds *M_B_* (Fig. 3 ▶, green curve). This behaviour further confirms the fact that, beyond the range of their reliable definition, the Shannon channels do not significantly improve the fit by the interpolated curve but, at the same time, they lead to strong oscillations in the *p*(*r*) function (clearly seen in Fig. 2 ▶ *b*). The target function *f*(*M*) is dominated by the discrepancy term χ^2^(*M*) (misfit to the data) at smaller *M*, and by the rapidly increasing integral derivative Ω(*p*_*M*_), due to an oscillating *p*_*M*_(*r*) function at larger *M* (Fig. 3 ▶, red curve). This leads to a characteristic U-shaped profile of *f*(*M*) and allows for a straightforward localization of *M*_S_ corresponding to the minimum of the target function.

A computer program, *Shanum*, was written to perform the selection of *M*_S_ following the above algorithm. To verify its performance, *Shanum* was applied to the simulated scattering curves described in the previous section, and it determined *M*_S_ values coinciding with *M_B_* within one Shannon channel for all cases (Table 1 ▶). These extensive test calculations indicated that the proposed algorithm allows one to determine reliably the effective number of Shannon channels in a data set *M*_S_ and therefore the useful range of the experimental data (since *s* = π*M*_S_/*D*_max_).

## 5. Examples of practical application   

**Figure 4:**
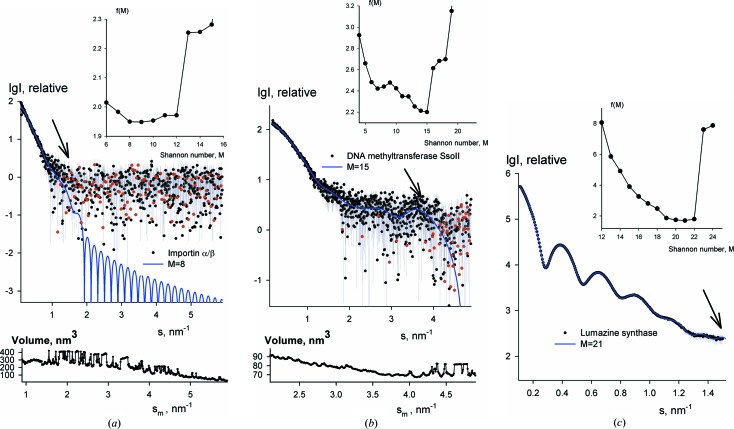
Experimental SAXS data and Shannon fits corresponding to the optimum number of channels determined by *Shanum*. (*a*) The complex of Importin α/β (Falces *et al.*, 2010 ▶). (*b*) The M.SSoII protein (Konarev *et al.*, 2014 ▶). (*c*) The LSAQ-IDEA protein (Zhang *et al.*, 2006 ▶). The useful angular range is identified by an arrow in each case and the target function is shown in the inset. The insets at the bottom of parts (*a*) and (*b*) display the dependence of the hydrated particle (Porod) volume on the range of experimental data used for the calculation of the Porod invariant (see *Discussion* section).

After validation using simulated data, the method was applied to a number of experimental X-ray and neutron data sets collected over different angular ranges from macromolecular solutions containing particles of various sizes at different concentrations. Some of these examples are presented below to illustrate the capacity of the method to detect the useful data range. The X-ray synchrotron scattering data were recorded in collaborative user projects on the X33 beamline of the EMBL (Blanchet *et al.*, 2012 ▶) at the storage ring DORIS-III (DESY, Hamburg). Fig. 4 ▶(*a*) presents the X-ray scattering data from an Importin α/β complex with a molecular mass (*M*_r_) of 160 kDa and *D*_max_ = 19 nm (Falces *et al.*, 2010 ▶). Due to the low protein concentration (0.5 mg ml^−1^), the scattering data are extremely noisy at higher angles. Despite the fact that the measured range of scattering vectors (up to *s*_max_ = 6 nm^−1^) nominally contains *N*_S_ = 36 Shannon channels, the algorithm returns *M*_S_ = 9, indicating that the high-angle data beyond *s* = 1.5 nm^−1^ contain no useful information. The scattering pattern from the DNA methyltransferase SsoII (*M*_r_ = 45 kDa, *D*_max_ = 11 nm) displayed in Fig. 4 ▶(*b*) (Konarev *et al.*, 2014 ▶) appears rather noisy starting from *s* = 2 nm^−1^, but the algorithm indicates that the data contain useful information up to 4 nm^−1^. The data from LSAQ-IDEA Lumazine synthase (Zhang *et al.*, 2006 ▶), which forms icosahedral assemblies in solution (with *M*_r_ = 2 MDa and *D*_max_ = 33 nm), display a good signal-to-noise ratio over the entire range displayed in Fig. 4 ▶(*c*) and the algorithm does find the full data range, with 20 Shannon channels to contain useful information. Interestingly, the *Shanum* estimates correlate well with the data ranges actually used for data analysis in the above-mentioned publications.

**Figure 5:**
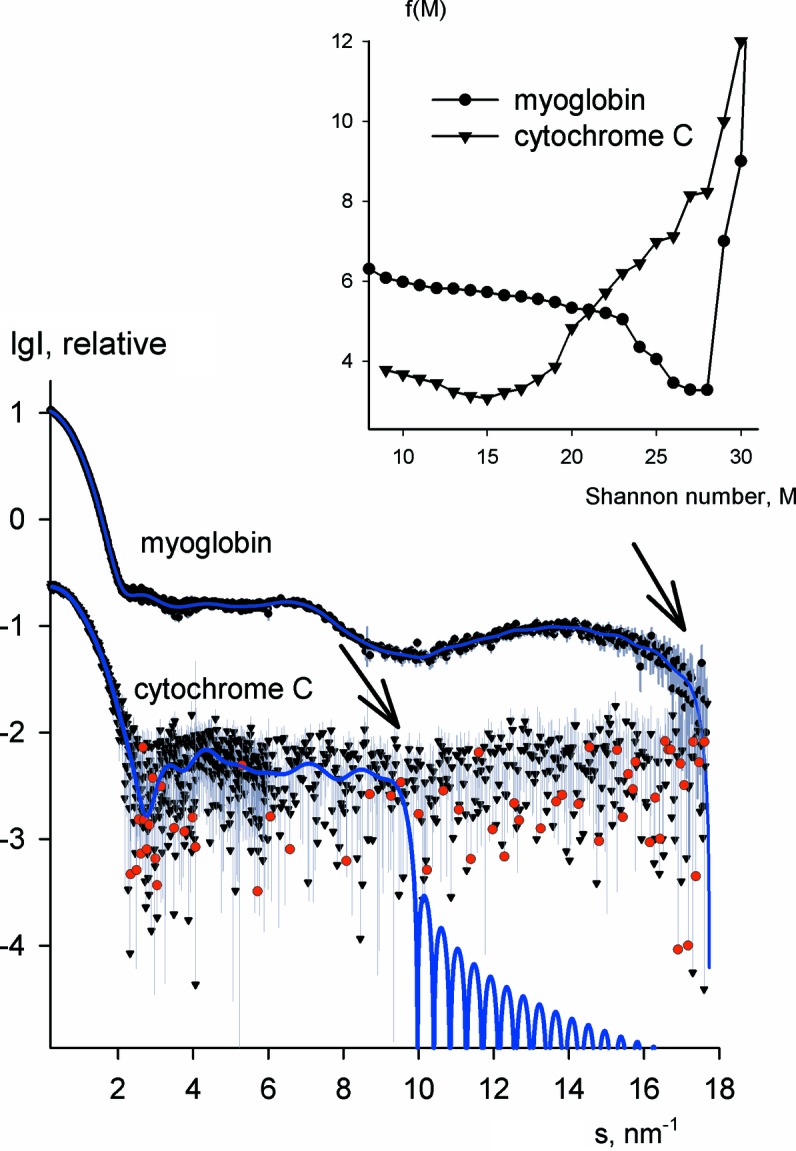
Experimental WAXS data and Shannon fits corresponding to the optimum number of channels determined by *Shanum*. The top curve is from a concentrated solution of myoglobin and the bottom curve is from a dilute solution of cytochrome *c*. The useful angular ranges are identified by the arrows and the target functions are shown in the inset.

It was also interesting to check whether the method is applicable to wide-angle X-ray scattering (WAXS) data. WAXS curves provide higher-resolution information and generally contain larger numbers of Shannon channels compared with SAXS data. We applied *Shanum* to WAXS data from a concentrated (28 mg ml^−1^) solution of myoglobin [downloaded from the Small-Angle Scattering Data Bank (SASBDB), www.sasbdb.org, entry SASDAK2] and from a dilute (2 mg ml^−1^) solution of cytochrome *c* (recorded at X33; unpublished data). Whereas for the former case the entire measured WAXS range was selected as useful, only about half of this range was deemed informative for the latter case (Fig. 5 ▶).

**Figure 6:**
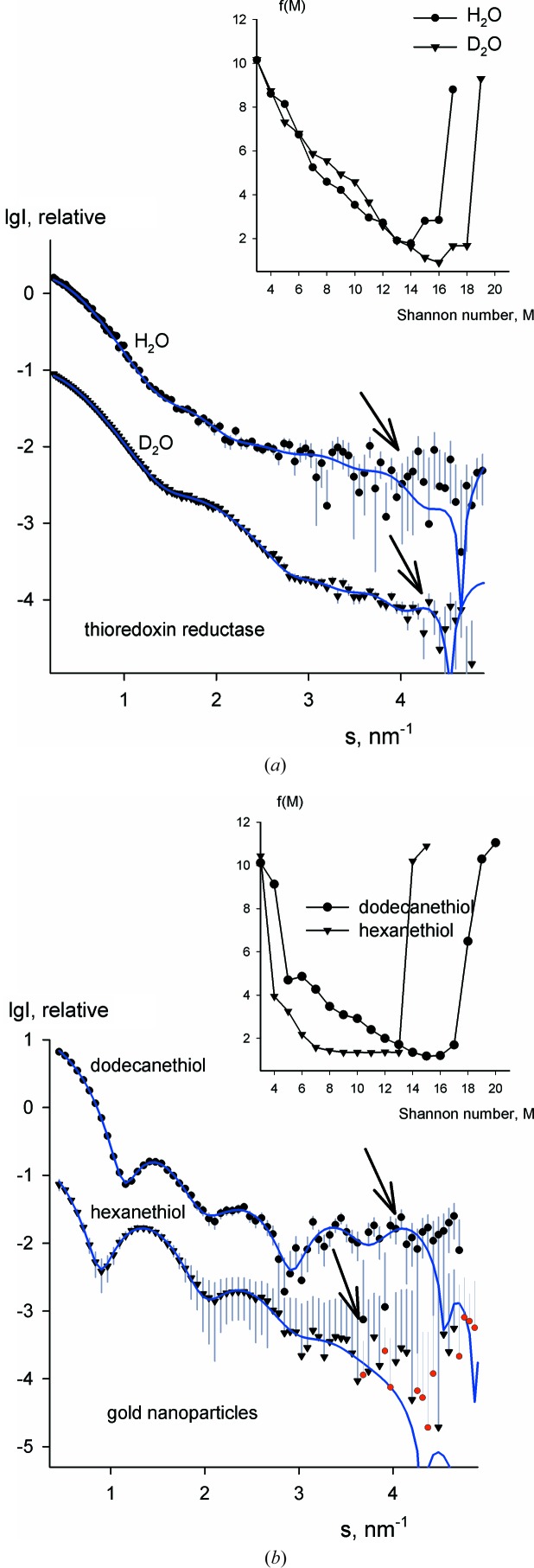
Experimental SANS data and Shannon fits corresponding to the optimum number of channels determined by *Shanum*. (*a*) Data from thioredoxin reductase solutions. The top and bottom curves are measured in H_2_O and D_2_O, respectively (Svergun *et al.*, 1998 ▶). (*b*) Scattering from composite gold nanoparticles in deuterated chloroform, with specifically deuterated dodecanethiol (upper curve) and hexanethiol (lower curve) (Moglianetti *et al.*, 2014 ▶). The useful angular ranges are identified by the arrows and the target functions are shown in the inset.

Finally, we shall illustrate the use of the algorithm on several published neutron scattering data sets. Fig. 6 ▶(*a*) displays SANS data from thioredoxin reductase, a dimeric protein with *M*_r_ = 68 kDa and *D*_max_ = 11 nm, recorded on the D22 instrument at the Institute Laue Langevin, Grenoble, France (Svergun *et al.*, 1998 ▶). The two data sets, collected in H_2_O and in D_2_O over the same angular range (up to *s*_max_ = 5.2 nm^−1^), nominally both cover *N*_S_ = 17 Shannon channels. However, the H_2_O data are noisier, due to the lower contrast and the incoherent background, such that the algorithm returns 14 effective channels for the H_2_O data and 16 channels for the D_2_O data. The next example demonstrates that the approach is not limited to biological macromolecules in aqueous solutions. The SANS data in Fig. 6 ▶(*b*) were collected on the KWS-2 beamline (Julich Centre for Neutron Science, FRM-II reactor, TU München, Germany) from hybrid gold nanoparticles protected by dodecanethiol (C_12_) or hexanethiol (C_6_) dissolved in deuterated chloroform (Moglianetti *et al.*, 2014 ▶). The top and bottom curves were recorded on the hybrid particles with specifically deuterated dodecanethiol or hexane­thiol, respectively. The composite nanoparticle solutions are close to monodisperse, with a diameter of 8 nm, as shown by the shapes of the scattering curves and also by complementary methods. *Shanum* provides feasible results, suggesting that most of the dodecanethiol curve is informative, whereas the last third of the noisier hexanethiol curve bears no useful information. Given that chemically synthesized nanoparticles inevitably have a certain degree of polydispersity, the presented example indicates the applicability of *Shanum* not only for a non-biological system but also for a slightly polydisperse one.

## 6. Discussion and conclusions   

Until now, no established procedure was available to assess the useful range of experimental SAXS and SANS data. The main problems of assessment based on the signal-to-noise ratio are a lack of objectivity in the selection of the threshold and the fact that the degree of oversampling is not taken into account. The proposed method overcomes both problems and offers an objective procedure to determine the useful range. The procedure, implemented in the program module *Shanum* included in the *ATSAS* package (http://www.embl-hamburg.de/biosaxs/software.html), is freely available to academic users, together with other *ATSAS* programs as from the 2.6 release.

Given an experimental data set, the program requires only the maximum size of the particle, *D*_max_, to determine the useful range. By default, the programs *AutoRG* and *AutoGnom* (Petoukhov *et al.*, 2007 ▶) are employed to estimate *D*_max_, but if this value is known *a priori* (*e.g.* when analysing data from a protein with a known structure) it can be specified by the user. Importantly, the Shannon formalism [equations (4)–(6)] is valid not only for the maximum size *D*_max_ but also for any value *D* > *D*_max_. This makes the entire procedure even more robust, allowing one safely to use a somewhat overestimated maximum size and also to handle slightly polydisperse systems (see the nanoparticle example presented above). In the test and practical calculations presented in this work, the use of 5–10% overestimated values yielded practically the same useful data ranges.

In X-ray crystallography, the useful data range assessed by *I*/σ and *R*_merge_ determines the set of reflections to enter the refinement and therefore directly defines the resolution of the model. In SAS, cutting out higher-angle data would not influence the accuracy of some parameters, *e.g.* the radius of gyration determined from low-angle data by the Guinier approximation (Guinier, 1939 ▶). Obviously, the removal of meaningless data is expected to improve the results of indirect transformation analysis and of the fitting procedures making use of WAXS data (*e.g.* shape determination using *GASBOR*; Svergun *et al.*, 2001 ▶), and also of the calculation of overall particle parameters such as the Porod volume *V*_p_. This last represents the excluded particle volume and is computed as (Porod, 1982 ▶) 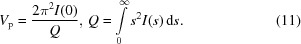
In practical applications, the Porod invariant *Q* is calculated over a finite range [0, *s*_*m*_] and appropriate corrections are applied to compensate for the missing data from *s*_*m*_ to infinity (*e.g.* in the *POROD* module of *PRIMUS*; Konarev *et al.*, 2003 ▶). The lower panel of Fig. 4 ▶(*a*) presents the Porod volume of the Importin α/β complex as a function of the upper integration limit *s*_*m*_. Given an empirical relation *V*_p_ (in nm^3^) ≃ 1.7–1.8*M*_r_ (in kDa) (Petoukhov *et al.*, 2012 ▶), the expected Porod volume of the complex is about 280 nm^3^. The volume computed directly by the *POROD* module provides stable values, with moderate variations in the useful data range detected by *Shanum* (*i.e.* up to *s*_*m*_ ≃ 1.3 nm^−1^), and starts to oscillate wildly as soon as higher-angle data are taken into account. Similarly, for DNA methyltransferase SsoII, *V*_p_ reveals meaningful values of around 75 nm^3^ when *s*_*m*_ stays within the useful data range and unreasonable oscillations beyond this range (lower panel of Fig. 4 ▶ *b*). Note that, in practice, the above empirical relation is used in the opposite direction and *V*_p_ is considered to be one of the ways of assessing *M*_r_ without absolute calibration. These examples illustrate the importance of the removal of meaningless data for preventing potential problems in the determination of basic particle parameters.

We should underline that the proposed algorithm is not intended to serve as a low-pass filter to provide noise reduction by fitting of the experimental data. As evident from Fig. 1 ▶, at high noise levels the Shannon fits may display noticeable artificial oscillations, especially for anisometric particles. Further, the truncated Shannon representations inevitably display a termination effect due to the missing higher orders [in particular, *U*_*M*_(*s*) exhibits unphysical negative values oscillating around zero for arguments exceeding π*M*/*D*_max_]. The method is developed as a means of assessing the information content, and not as a smoothing tool for noisy data.

In cases where the experimental errors in the data set are not available and the value of χ^2^ cannot be reliably calculated, one can use a recently developed correlation map test instead (Franke *et al.*, 2015 ▶). In this approach, the agreement between the experimental data and the Shannon approximation is measured by the longest contiguous stretch of the same sign of the residuals, whereby the length of this stretch can be translated into a statistical probability value. We have implemented the correlation map criterion in *Shanum* as an alternative to χ^2^ in equation (5) and found similar results to the use of discrepancy, allowing one to evaluate reliably the range of useful data also when the experimental errors are not available. The present version of *Shanum* uses the correlation map if the associated errors are not provided in the input experimental data set.

Importantly, the method proposed here does not require user input and is thus applicable in automated pipelines for data analysis. Further, *Shanum* is being implemented in a suite of validation tools for the deposited experimental SAXS/SANS data in SASBDB. The principle of assessment of the useful data range proposed here might be useful for other types of scattering or spectroscopic experiments yielding discrete oversampled data.

## Supplementary Material

Supporting information file. DOI: 10.1107/S2052252515005163/fs5097sup1.pdf

## Figures and Tables

**Table 1:** Tests on simulated data sets calculated from geometric bodies The theoretical scattering was calculated up to a momentum transfer value of *s*_max_ = 4nm^1^, and various noise levels (ranging from 1 to 400%) were added. The columns on the right-hand side from the nominal Shannon number *N*_S_ = *s*_max_ *D*_max_/ display the optimum number of Shannon channels *M*_*B*_ that provides the best agreement with the ideal (noise-free) curve. In each case, the maximum noise level where useful information is still present in the entire curve is shown in bold.

			*M*_*B*_						
Type of body	*D*_max_ (nm)	*N*_S_	1%	5%	20%	50%	100%	200%	400%
(*a*) X-ray type data, strong oversampling									
Oblate ellipsoid (half-axes 15, 15, 1nm)	30	38	41	39	39	38	38	**38**	25
Prolate ellipsoid (half-axes 1, 1, 15nm)	30	38	39	39	39	38	**38**	37	23
Hollow sphere (*R*_in_ 2.5nm, *R*_out_ 5nm)	10	13	14	14	13	13	**13**	12	11
Hollow cylinder (*R*_in_ 2.5nm, *R*_out_ 5nm, *H* 10nm)	14	18	19	19	18	18	**18**	16	14
Cube (5nm edge)	8.6	11	12	12	12	11	**11**	10	9
Solid sphere (radius 5nm)	10	13	15	14	14	14	14	14	**13**
									
(*b*) Neutron type data, medium oversampling									
Oblate ellipsoid (half-axes 15, 15, 1nm)	30	38	38	38	38	38	**38**	37	25
Prolate ellipsoid (half-axes 1, 1, 15nm)	30	38	38	38	**38**	37	36	34	15
Hollow sphere (*R*_in_ 2.5nm, *R*_out_ 5nm)	10	13	13	13	**13**	12	11	11	10
Hollow cylinder (*R*_in_ 2.5nm, *R*_out_ 5nm, *H* 10nm)	14	18	18	18	**18**	17	16	16	14
Cube (5nm edge)	8.6	11	11	11	**11**	10	10	9	8
Solid sphere (radius 5nm)	10	13	13	13	13	13	**13**	11	10
