# Supplementary material for: A posteriori determination of the useful data range for small-angle scattering experiments on dilute monodisperse systems
Source: IUCrJ. 2015 Apr 21;2(Pt 3):352–60. doi: 10.1107/S2052252515005163 (PMC4420545; doi:10.1107/S2052252515005163)
Supplement: Supplementary file 1 [file m-02-00352-sup1.pdf]

IUCrJ

Volume 2 (2015)

Supporting information for article:

A posteriori determination of the useful data range for small-angle scattering experiments on dilute monodisperse systems

Petr V. Konarev and Dmitri I. Svergun

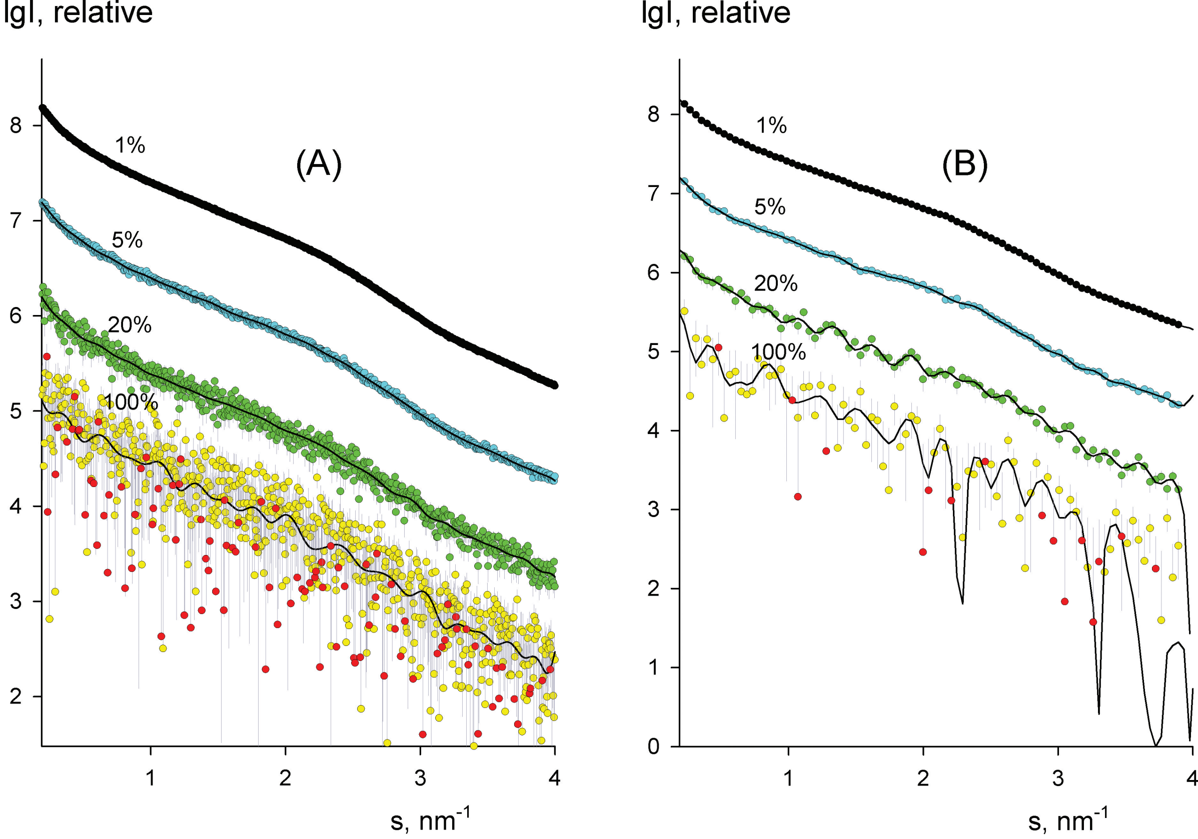

Figure S1 Simulated scattering curves from a prolate ellipsoid (half-axes 1, 1 and 15 nm). Curves 1 to 4 correspond to added Gaussian noise of 1, 5, 20 and 100 % (dots with error bars). The best truncated Shannon approximations are displayed as solid lines. The subsequent curves are shifted by one logarithmic order for better visualization. Panel A corresponds to typical X-ray type data, panel B to typical neutron type data.

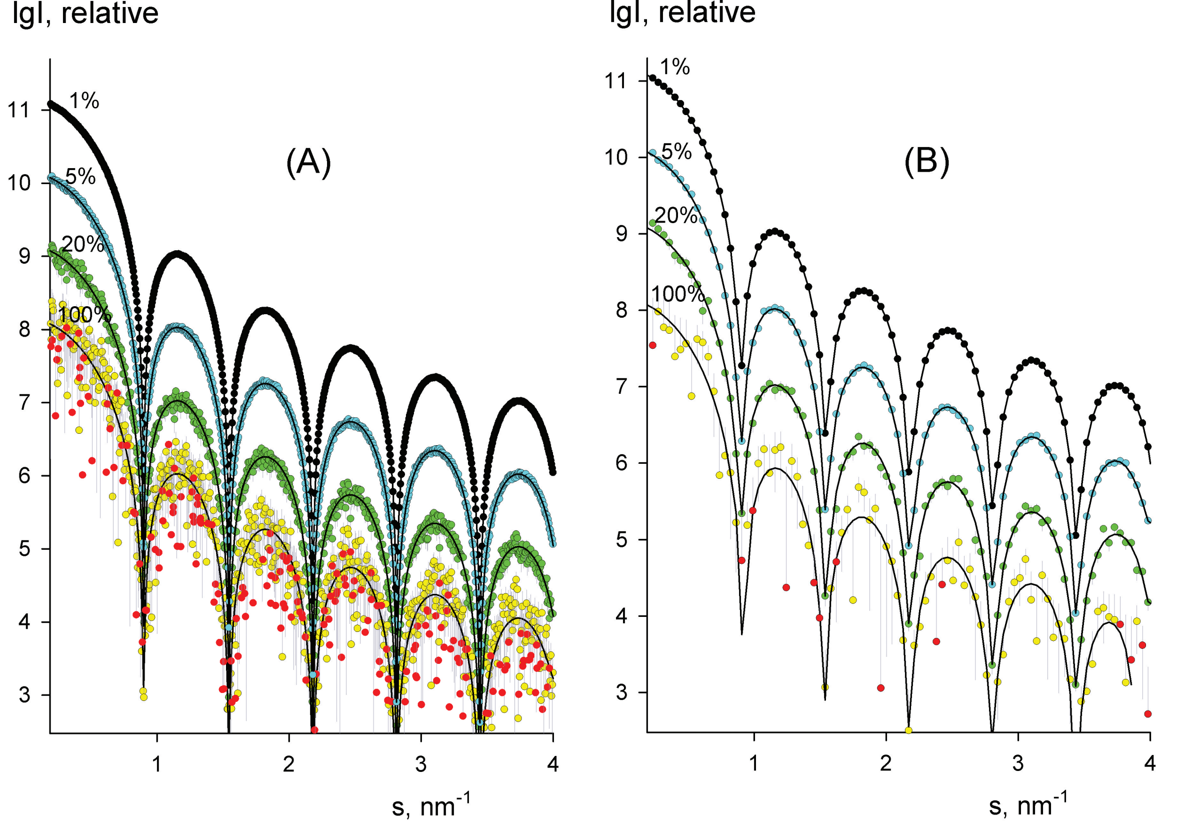

Figure S2 Simulated scattering curves from a sphere (radius 5 nm). Curves 1 to 4 correspond to added Gaussian noise of 1, 5, 20 and 100 % (dots with error bars). The best truncated Shannon approximations are displayed as solid lines. The subsequent curves are shifted by one logarithmic order for better visualization. Panel A corresponds to typical X-ray type data, panel B to typical neutron type data.

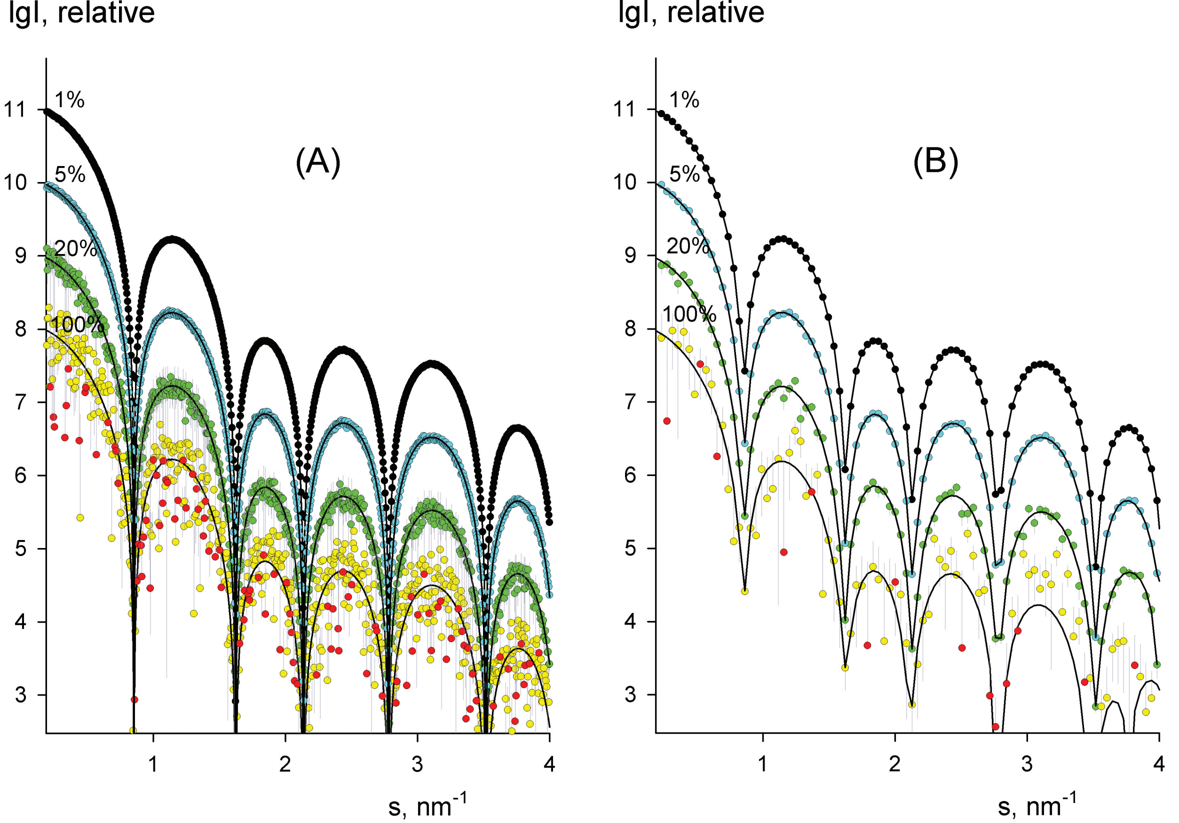

Figure S3 Simulated scattering curves from a hollow sphere (2.5 nm inner radius, 5nm outer radius). Curves 1 to 4 correspond to added Gaussian noise of 1, 5, 20 and 100 % (dots with error bars). The best truncated Shannon approximations are displayed as solid lines. The subsequent curves are shifted by one logarithmic order for better visualization. Panel A corresponds to typical X-ray type data, panel B to typical neutron type data.

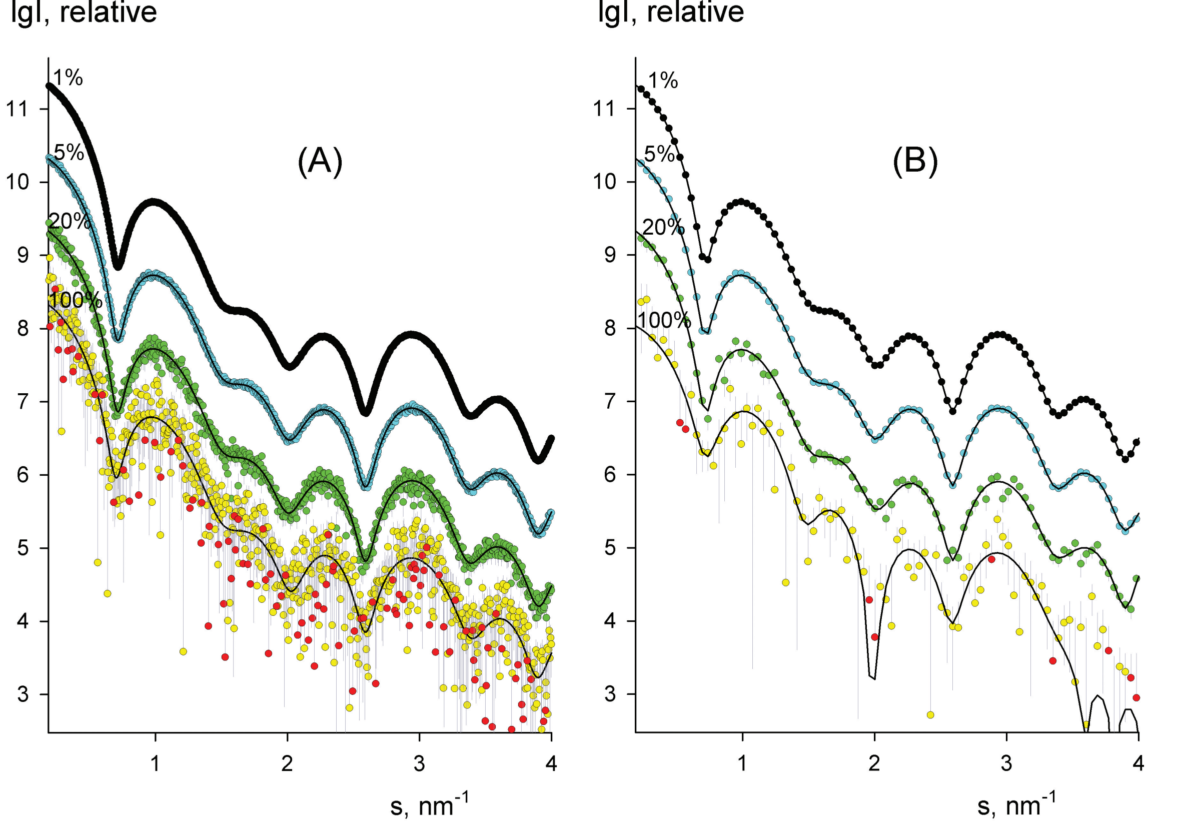

Figure S4 Simulated scattering curves from a hollow cylinder (2.5 nm inner radius, 5 nm outer radius, 10 nm height). Curves 1 to 4 correspond to added Gaussian noise of 1, 5, 20 and 100 % (dots with error bars). The best truncated Shannon approximations are displayed as solid lines. The subsequent curves are shifted by one logarithmic order for better visualization. Panel A corresponds to typical X-ray type data, panel B to typical neutron type data.
